# Residents’ perspective on the quality of postgraduate training programs in Pakistan – the good, the bad and the ugly

**DOI:** 10.12669/pjms.37.7.4297

**Published:** 2021

**Authors:** Laima Alam, Jawad Khan, Mafaza Alam, Varqa Faraid, Fahad Ajmal, Laila Bahadur

**Affiliations:** 1 Laima Alam, FCPS Gastroenterology, MRCP. Bahria Town International Hospital, Rawalpindi, Pakistan; 2 Jawad Khan, FCPS Pulmonology, FCPS CCM. Bahria Town International Hospital, Rawalpindi, Pakistan; 3 Mafaza Alam, Registrar Operative Dentistry, AFID, Rawalpindi, Pakistan; 4 Varqa Faraid SZABMU/School of Dentistry, Islamabad, Pakistan; 5 Fahad Ajmal, FCPS Medicine, SR CCM. Bahria Town International Hospital, Rawalpindi, Pakistan; 6 Laila Bahadur, MPhil, FCPS Clinical Hematology, Bahria Town International Hospital, Rawalpindi, Pakistan

**Keywords:** Clinical skills, Medical education, Postgraduate training, Quality of training Survey

## Abstract

**Objectives:**

To assess the satisfaction of trainees towards different attributes of their training programs.

**Methods:**

This cross-sectional survey was carried out by enrolling trainee doctors currently working in Medical, Surgical, Dental and Allied specialties of the country by sending a validated and piloted questionnaire through email. Data collection was done from 1^st^ to 31^st^ January 2021 after taking ethical approval from the concerned authorities. Data was analysed using SPSS v. 19.0.

**Results:**

A total of 516 completed responses were received from 15 major cities of the country. The overall perceived satisfaction towards clinical skills (42%), teaching skills (31.4%), personal growth and development (23.6%), research (21%) and supervisor’s role (44.2%) were considerably low with the most common causes for non-satisfaction being poor work-life balance (59%), financial instability (54.5%), poor research facilities (53%), poor career guidance (44%) and poor skill development (42.4%) in descending order. Senior years of residency, government and private set-ups, less than four and greater than 13 residents on average with less than three supervisors per department, excessive duty hours and financial instability in-lieu of not doing locums were statistically related to poor satisfaction across majority of the facets of residency as well the overall satisfaction towards training programs.

**Conclusion:**

There is a tremendous scope for improvement in the recognized and partially acknowledged attributes of our training programs. Yearly feedback surveys involving residents is essential for enlightening the authorities and mitigating the trainees’ grievances.

## INTRODUCTION

The objective of medical residency programs has moved from the bare minimal standard attainment to an elaborate system of continuous improvement with frequent appraisals and evaluations for validation.^1^ Accreditation of a highly robust residency program involves work environment, academics and its balance with service, evaluations , mentorship and the qualities of the supervisors.^2^ Frequent scientific evaluation of training programs has long been advocated to ensure safe and productive environment for the residents that in turn affects performance and adequate patient care.^3^

Every medical education program, be it under-graduate or post-graduate, requires a system of continuous analysis, policy making and reassessing the improvements brought about by the implemented strategies.^4^ Unfortunately, there is a serious dearth of such quintessential research in our country, leading to uneven quality of training with rising stress, maladaptive coping strategies and feeling of abandonment in majority of the trainee doctors. An extensive literature review showed that only a few studies were conducted to identify unrecognized deficiencies of our training programs and that too were either geographically limited to a single centre, city or a province or a single specialty^3^,^5^-^7^, leading to results that could not be generalized.

This nation-wide cross-sectional survey was designed to measure the level of satisfaction of our residents working in varied set-ups towards various facets of residency that were not previously studied locally. The relation of multiple demographic variables with satisfaction scores was also studied in detail.

## METHODS

This cross-sectional survey was carried out by enrolling trainee doctors working in different cities of Pakistan through convenience sampling after acquiring ethical approval from the concerned department (A/01/21/13, dated January 1, 2021). The survey was completed in one month i.e.; from 1^st^ January 2021 to 31^st^ January 2021 by enrolling trainee doctors currently working in Medical, Surgical, Dental and Allied specialties of the country. Trainees from basic medical sciences, non-trainee doctors and those with less than six months experience were all excluded.

The questionnaire was developed by LA, MA and JK after a thorough literature review 5-8 and was reviewed by two medical education experts for content validity. The survey was piloted among 10 post-graduate residents before putting it to test. The questionnaire encompassed perceived satisfaction towards six main facets of residency programs including clinical skills, teaching, personal growth and development, research, supervisor’s role and environment of the training institute.^9^ The perceived quality was scored using Likert five-point scale ranging from strongly agree (1) to strongly disagree (5). Questions regarding each domain of the residency programs were followed by a Yes/No question in order to enable the participants to select their over-all satisfaction regarding that domain.

The sample size was calculated with margin of error set at 4.5%, confidence level at 95% and an anticipated frequency (response distribution) of 50% using OpenEpi sample size calculator. The questionnaire was sent through email, a reminder was given to the participants after one week of no response and the candidates were dropped who failed to respond after another seven days.^10^

### Statistical Analysis

To measure the internal consistency of the instrument, Cronbach’s alpha was calculated which produced a value of 0.95. Qualitative data was expressed as frequencies and percentages. Relation of non-satisfaction with socio-demographic variables was seen using multinomial logistic regression. A value of <0.05 was considered statistically significant. All analysis was done using SPSS V.19.

## RESULTS

**Fig.1:**
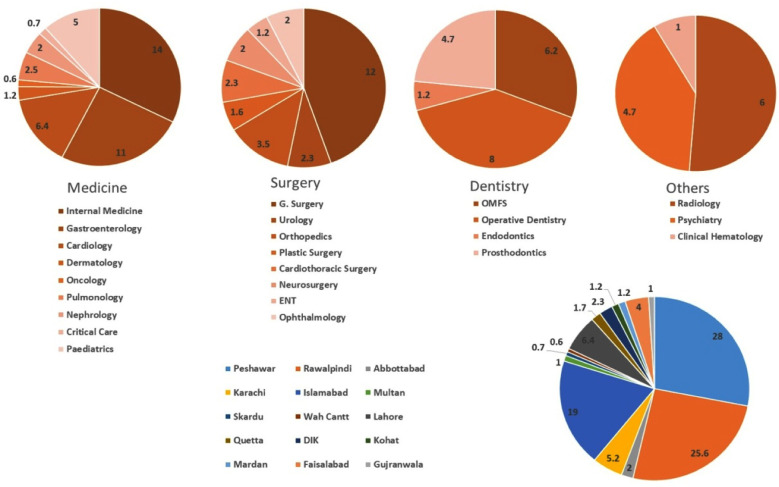
Distribution of different specialties and cities of residency.

A total of 516 completed responses were received (from 15 major cities of the country representing all the provinces as shown in Figure 1) out of 960 emails sent, making a response rate of 53.7%. Male participants (65.7%) with a median age of 24-30years (63%) getting trained at government (47%) followed by army set-up (34.4%) showed maximum participation. An average of 26-30 working days (51.7%) with 4-6 on-calls (28%), 1-3 long-days (22.7%) and 1-2 weekends-on-call (70%) was the rota per month for majority of the trainees (Table-I).

**Table I:** Demographics of the participants enrolled

*Variables*	*Frequency*	*Percentage*
***Gender*** (M/F)	339/177	65.7/34.3
***Age*** (years) 24-30/31-35/36-40/41-45	324/180/6/6	62.8/34.9/1.2/1.2
***Marital status*** (Single/Married)	228/288	44.2/55.8
***Family size*** (<5/5-8/>8)	279/183/54	54.1/35.5/10.5
***No of dependents*** None/1-3/4-6/>6	177/207/96/36	34.3/40.1/18.6/7
***Year of residency*** 1^st^/2^nd^/3^rd^/4^th^/5^th^	84/93/69/171/99	16.3/18/13.4/33.1/19.2
***Nature of set-up*** Government/Private/Army	243/96/177	47/18.6/34.3
***Total no. of residents in the department*** <4/4-7/8-12/13-16/17-20/21-24/25-28/>28	51/102/39/81/60/72/69/42	9.9/19.8/7.6/15.7/11.6/14/13.4/8.1
***Total no. of supervisors*** 1/2/3/4/5/>5	159/84/159/39/21/54	30.8/16.3/30.8/7.6/4.1/10.5
***No. of International Medical Graduates as residents*** None/1-3/>3	429/45/42	83.1/8.7/6.4
***Monthly take home salary*** (Rs) <30,000/30,000-50,000/50,000-80,000/>80,000	138/52/156/156	26.7/10/30.2/30.2
***Locum***(Yes/No)	192/324	37.2/62.8
***Strong ties with the city of residency*** (Yes/No)	318/198	61.6/38.4
***No. of work days per month*** 15-20/21-25/26-30	45/204/267	8.7/39.5/51.7
***No. of on-calls per month*** None/1-3/4-6/7-9/10-13/≥14	66/129/144/90/60/27	12.8/25/27.9/17.4/11.6/5.2
***No. of weekend-on-calls per month*** (0/1-2/3-4)	114/360/42	22/70/8
***No. of long days per month*** None/1-3/4-6/7-9/10-13/≥14	114/117/105/69/51/60	22.1/22.7/20.3/13.4/9.9/11.6

**Fig.2:**
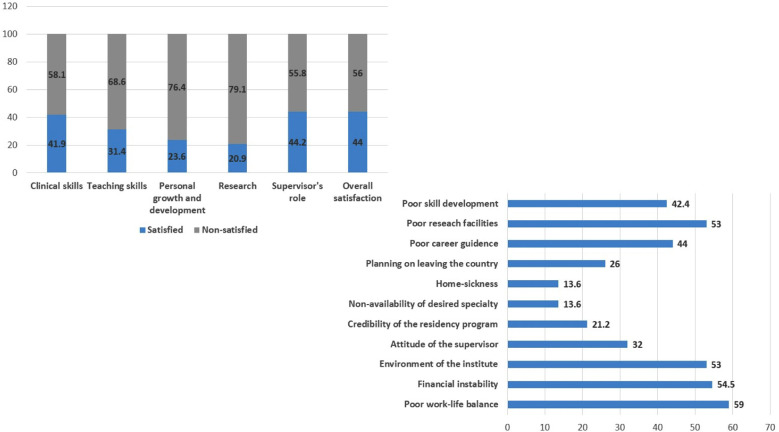
Overall satisfaction of the trainees with reasons for non-satisfaction towards various facets of training.

The scores of perceived satisfaction using a five-point scale for different facets of training is shown in Table-II. The overall perceived satisfaction towards clinical skills (42%), teaching skills (31.4%), personal growth and development (23.6%), research (21%) and supervisor’s role (44.2%) were considerably low with the most common causes for non-satisfaction being poor work-life balance (59%), financial instability (54.5%), poor research facilities (53%), poor career guidance (44%) and poor skill development (42.4%) in descending order (Figure 2).

**Table II:** Assessment of the quality of training programs using five-point scale.

*As per CPSP recommendation, are you provided with/facilitated in:*	*Strongly agree*	*Agree*	*Neutral*	*Disagree*	*Strongly disagree*
***Clinical skills***					
Hands on	84(16.3)	123(23.8)	153(29.7)	51(9.9)	105(20.3)
Elective rotations	90(17.4)	81(15.7)	93(18)	69(13.4)	183(35.5)
Adequate OPD patient exposure	240(46.5)	105(20.3)	78(15.1)	30(5.8)	63(12.2)
Adequate OT/procedure room exposure	105(20.3)	132(25.6)	99(19.2)	66(12.8)	114(22.1)
Adequate supervision during procedures	72(14)	120(23.3)	138(26.7)	57(11)	129(25)
Adequate exposure to advance procedures	39(7.6)	132(25.6)	138(26.7)	66(12.8)	141(27.3)
Adequate direct/indirect supervision	63(12.2)	105(20.3)	168(32.6)	75(14.5)	105(20.3)
Mandatory workshop	102(19.8)	162(31.4)	111(21.5)	69(13.4)	72(14)
Adequate range of pathology and patient volume	69(13.4)	117(22.7)	177(34.3)	42(8.1)	111(21.5)
***Teaching skills***					
Attending/presenting MDT	63(12.2)	99(19.2)	99(19.2)	165(32)	90(17.4)
Presenting/ attending clinical presentation, morning meetings, CBDs, CPC etc.	162(31.4)	165(32)	72(14)	84(16.3)	33(6.4)
Receiving teaching sessions by consultants	54(10.5)	150(29.1)	102(19.8)	102(19.8)	108(20.9)
Teaching sessions by trainees to juniors	81(15.7)	183(35.5)	123(23.8)	84(16.3)	45(8.7)
***Personal growth and development***					
Recommendations and experience certificates	69(13.4)	84(16.3)	177(34.3)	87(16.9)	99(19.2)
Attending in-person medical conferences	45(8.7)	93(18)	144(27.9)	90(17.4)	144(27.9)
Evaluations and appraisals	33(6.4)	75(14.5)	162(31.4)	132(25.6)	114(22.1)
Does your institute provide BLS and ACLS accreditation	69(13.4)	93(18)	105(20.3)	69(13.4)	180(34.9)
Acquiring CMEs and maintaining a portfolio	39(7.6)	75(14.5)	138(26.7)	132(25.6)	132(25.6)
Are you able to balance work and personal life	39(7.6)	87(16.9)	144(27.9)	129(25)	117(22.7)
Does your program have the ability to encourage and support life-long learning?	45(8.7)	105(20.3)	138(26.7)	102(19.8)	126(24.4)
Does your program have the ability to meet its recommended goals?	45(8.7)	147(28.5)	153(29.7)	81(15.7)	90(17.4)
***Research***					
A dedicated research unit	24(4.7)	66(12.8)	123(23.8)	96(18.6)	207(40.1)
Planning and execute audits/quality improvement projects	57(11)	60(11.6)	114(22.1)	120(23.3)	165(32)
Database, seminars/teaching sessions and help with topic selection	33(6.4)	51(9.9)	138(26.7)	123(23.8)	171(100)
Funding from institute	18(3.5)	30(5.8)	69(13.4)	153(29.7)	246(47.7)
Presenting papers/posters	42(8.1)	117(22.7)	129(25)	96(18.6)	132(25.6)
Protected academic or research time per week	30(5.8)	24(4.7)	132(25.6)	129(25)	201(39)
***Supervisor’s role***					
Adequate time spent in weekly clinical activities by the supervisors	39(7.6)	123(23.8)	111(21.5)	96(18.6)	147(28.5)
Adequate time spent in weekly research activities	12(2.3)	111(21.5)	108(20.9)	105(20.3)	180(34.9)
Adequate supervision by the faculty	45(8.7)	93(18)	114(22.1)	114(22.1)	150(29.1)
Adequate clinical skills of the faculty	117(22.7)	186(36)	105(20.3)	30(5.8)	78(15.1)
Revalidation and assessment programs for the supervisors	30(5.8)	126(24.4)	132(25.6)	48(9.3)	180(34.9)
Are your training supervisor and administrative office well informed of residents’ issues?	54(10.5)	123(23.8)	129(25)	90(17.4)	120(23.3)
Are your training supervisor and administrative office responsive to residents’ issues?	45(8.7)	108(20.9)	135(26.2)	81(15.7)	147(28.5)
***Others***					
Does your institute provide Hospital accommodation?	99(19.2)	105(20.3)	96(18.6)	60(11.6)	156(30.2)
Do you have an adequately functioning Cafeteria?	132(25.6)	111(21.5)	126(24.4)	51(9.9)	96(18.6)
Do you have an adequately functioning doctor’s room?	108(20.9)	120(23.3)	120(23.3)	60(11.6)	108(20.9)
Do you get paid for extra working hours?	0	30(5.8)	27(5.2)	9(1.7)	450(87.2)
Do you regularly receive patient feedback?	27(5.2)	69(13.4)	177(34.3)	87(16.9)	156(30.2)
Do you regularly receive peer feedback?	42(8.1)	39(7.6)	177(34.3)	108(20.9)	150(29.1)
Have you experienced workplace harassment?	84(16.3)	66(12.8)	75(14.5)	99(19.2)	192(37.2)
Have your peers experienced workplace harassment?	75(14.5)	102(19.8	117(22.7)	63(12.2)	159(30.8)
Do you have a workplace harassment monitoring and control disciplinary team?	21(4.1)	99(19.2)	111(21.5)	57(11)	228(44.2)
Is the atmosphere generally relaxed and not condescending?	39(7.6)	117(22.7)	159(30.8)	51(9.9)	150(29.1)
Are the residency programs essentially similar throughout the country?	27(5.2)	57(11)	93(18)	96(18.6)	243(47.1)
Is there any monitoring/evaluation available for your residency program?	27(5.2)	63(12.2)	162(31.4)	51(9.9)	213(41.3)

Senior years of residency, government and private set-ups, less than four and greater than 13 residents on average with less than three supervisors per department, excessive duty hours and financial instability in-lieu of not doing locums were statistically significant in relation to poor satisfaction across majority of the facets of residency as well the overall satisfaction towards training programs (Table-III).

**Table III:** Relation of demographics with overall satisfaction of the trainees using multinomial regression analysis.

*Variables*	*Clinical skills (p)*	*Teaching skills (p)*	*Personal growth and development (p)*	*Research (p)*	*Supervisor’s role (p)*	*Overall satisfaction (p)*
Gender	0.008	<0.001	0.05	0.09	0.08	0.07
Year of residency	0.05	<0.001	<0.001	<0.001	0.02	<0.001
Set-up	<0.001	<0.001	<0.001	<0.001	<0.001	<0.001
Low monthly salary^£^	0.03	0.92	0.55	<0.001	0.05	0.64
No of residents per department	<0.001	<0.001	<0.001	<0.001	<0.001	<0.001
No of supervisors per department	<0.001	0.012	<0.001	<0.001	<0.001	<0.001
Doing locum	<0.001	<0.001	<0.001	<0.001	0.01	<0.001
Excessive duty hours*	0.03	0.014	<0.001	<0.001	0.03	<0.001

£ <50,000 Rs

* ≥10 on-calls, >2 weekend-calls or ≥10 long days per month.

## DISCUSSION

An extensive analysis of our residency programs by Biggs JS in 2008 pointed out several short-comings including the lack of stipend for full time residents, poor career guidance with poor clinical skills procurement, disregard towards the laid-out curriculum, too many trainee doctors with too few mentors to supervise, lack of research and adequate hospital facilities and a serious dearth of evaluation of the residency programs.^11^ Although there is a clear national interest in training of doctors, hardly any improvement has been observed over more than a decade.

Job satisfaction is intrinsically linked to engagement and recognition, financial compensation (in the form of pay scale) and work-life balance^12^, all of which were unfortunately reported to be infringed in this study. About 27% of the trainees iterated a monthly salary of less than Rs 30,000 (187 USD) and 37% less than Rs 50,000 (314 USD) with 89% of the trainees reporting no financial compensation for extra duty hours. The financial constraints can be easily deduced from the fact that 56% of the trainees were married and had, on average, three or more dependents with only 37% managing to supplement their salary with locums.

Poor research skills and non-availability of funding or protected research hours were common for all the residents irrespective of the hospital set-up and is the facet that scored the lowest in terms of satisfaction in this study. Our residency programs need to bring about changes to help equip the trainees with agility to encourage life-long learning and to foster research culture.^13^

Revalidations for the supervisors and assessment of the training were not available for many of the residency programs in our study. An adequately trained supervisor is integral for an effective clinical program and learner’s autonomy and it is the responsibility of the College of Physicians and Surgeons of Pakistan to prepare their faculty for this role.^14^ Despite all the hurdles and resource limitations, the supervisors are doing their best to provide quality training and their role was the only facet in the current study that showed maximum satisfaction in comparison.

Moreover, 29-34% of the trainees reported workplace harassment either involving themselves or a colleague with only 23% of the institutions providing disciplinary committees to deal with these allegations. A study by Hu Y et al demonstrated that 59.4% of medical trainees had experienced at least one form of harassment or discrimination during their training with consultants being the most common perpretrator.^15^ The authors believe the percentage of work-place harassment might be under-reported due to the stigma attached to disclosure and, more importantly, many of our trainees usually don’t know what constitutes work-place harassment and bullying.^16^ There is a need for stringent policies and cultural change at our set-ups to provide a healthy progressive environment.

There was a common trend seen regarding non-satisfaction across all the facets of training that included working in public set-ups, greater number of residents per department versus lower number of supervisors for mentoring, excessive duty hours and financial instability in relation to not doing locums. The trainees working in army set-ups showed higher levels of satisfaction and reported better clinical skills with greater contentment towards supervisors’ role in their residency programs. On the contrary, >5 supervisors in a department were considered to be adversely affecting the clinical skills, likely because of less opportunity provided for hands-on and advanced skill procurement. Junior trainees were not happy with their supervisors whereas senior trainees showed a relatively higher non-satisfaction towards their clinical and teaching skills development, findings similar to a study conducted on Greek residents.^17^

Female trainees conveyed a statistically significant non-satisfaction towards their clinical and teaching skills which has been studied extensively in the past showing gender based discrimination in residency and practice.^18^ Male residents were not happy with their personal growth and development and the over-all non-satisfaction was related to <4 or >13 residents per department with <3 supervisors, higher residency year (likely secondary to the realization that the program failed a trainee’s initial expectations)^17^ and financial constraints through multinomial regression analysis.

It was interesting to see that for some of the survey questions the neutral response was as high as 34.3%, a trend observed in a similar local study.^5^ It has been seen that choosing a neutral option provides an easy out for the participants who are less willing to express their opinion or when they are reluctant to voice a socially disagreeable sentiment.^19^ This aloofness might be one of the biggest confounders responsible for the lack of prompting for conceivable policies and an imperative change.

Resident doctors’ burnout in lieu of poor organizational systems is one of the most notorious factors for eroding their wellness and affecting the patients’ quality of care and general satisfaction.^20^ Reflection, leadership, continuous monitoring and assessment with residents’ feedback are paramount for a cohesive and robust curriculum that has the ability to encourage and support life-long learning.

### Limitations of the Study

The only limitation of the study is simple convenience sampling.

## CONCLUSION

There is a tremendous scope for improvement in the recognized and partially acknowledged attributes of our training programs. Regular monitoring of the training programs along with repeated validation of the supervising mentors is mandatory for improved outcomes. Yearly feedback surveys involving residents is essential for enlightening the authorities and mitigating the trainees’ grievances.

### Author Contribution:

**LA, JK, MA:** Contributed to the idea, questionnaire and data collection.

**LA:** Contributed to the design, statistical analysis and drafting of the manuscript.

**VF, FA, LB:** Contributed to data collection.

All authors take equal responsibility for the accuracy and integrity of the work.
